# Cost-effectiveness of enzyme replacement therapy for type 1 Gaucher disease

**DOI:** 10.1186/1750-1172-9-51

**Published:** 2014-04-14

**Authors:** Laura van Dussen, Marieke Biegstraaten, Carla EM Hollak, Marcel GW Dijkgraaf

**Affiliations:** 1 Department of Internal Medicine, Division of Endocrinology and Metabolism, Academic Medical Center, PO Box 22660, 1100 DD Amsterdam, The Netherlands; 2 Clinical Research Unit, Academic Medical Center, PO Box 22660, 1100 DD Amsterdam, The Netherlands

## Abstract

**Objective:**

To evaluate the cost-effectiveness of enzyme replacement therapy (ERT) compared to standard medical care without ERT in the Dutch cohort of patients with type 1 Gaucher disease (GD I).

**Design:**

Cost-effectiveness analysis was performed using a life-time state-transition model of the disease’s natural course. Transition probabilities, effectiveness data and costs were derived from retrospective data and prospective follow-up of the Dutch study cohort.

**Setting:**

The tertiary referral center for Gaucher disease in the Netherlands.

**Participants:**

The Dutch cohort of patients with GD I.

**Intervention:**

ERT versus standard medical care without ERT in symptomatic patients.

**Main outcome measures:**

Years free of end organ damage (YFEOD) (splenectomy, bone complication, malignancy, multiple complications), quality adjusted life years (QALY), and costs.

**Results:**

Over an 85 year lifetime, an untreated GD I patient will generate 48.9 YFEOD and 55.86 QALYs. Starting ERT in a symptomatic patient increases the YFEOD by 12.8 years, while the number of QALYs gained increases by 6.27. The average yearly ERT medication costs range between €124,000 and €258,000 per patient. The lifetime costs of ERT starting in the symptomatic stage are €5,716,473 against €171,780 without ERT, a difference of €5,544,693. Consequently, the extra costs per additional YFEOD or per additional QALY are €434,416 and €884,994 respectively. After discounting effects by 1.5% and costs by 4% and under a reasonable scenario of ERT unit cost reduction by 25%, these incremental cost-effectiveness ratios could decrease to €149,857 and €324,812 respectively.

**Discussion:**

ERT is a highly potential drug for GD I with substantial health gains. The conservatively estimated incremental cost-effectiveness ratios are substantially lower than for Pompe and Fabry disease. We suggest that the high effectiveness has contributed importantly to acceptance of reimbursement of ERT for GD I. The present study may further support discussions on acceptable price limits for ultra-orphan products.

## Introduction

Orphan or rare diseases are life threatening or chronically debilitating, complex conditions with a prevalence of < 5 per 10.000. Over the last years, the interest of pharmaceutical companies in these disorders has grown, due to new legislation related to orphan drug development implemented in the US in 1983 and in Europe in 1999. New medications for orphan diseases, including many inherited metabolic disorders, have been developed. Clinical evaluation of newly developed products is sometimes difficult due to scarce knowledge and limited size of patients groups. Because of time constraints, trials often make use of surrogate endpoints that are not always clearly related to “hard” clinical endpoints such as death or the occurrence of serious complications that interfere with quality of life. As a consequence products are usually registered under ‘exceptional circumstances’, which implies that it is accepted that comprehensive data are unlikely to become available due to the rarity of the disorder.

Gaucher disease (GD; OMIM#230800), a very rare disorder with a prevalence of around 1 in 70.000, is a lysosomal storage disorder that results from defective activity of the lysosomal enzyme glucocerebrosidase (or acid β-glucosidase, EC 3.2.1.45). Storage of glucocerebroside in macrophages gives rise to hepatosplenomegaly, severely debilitating bone disease and, in rare cases, central nervous system involvement [1]. Three types of GD have been described. Type I GD (GD I) is the most common phenotype, and can be distinguished from the more severe types II and III GD based on the absence of the typical neurologic manifestations associated with the latter two forms [1,2]. Long term complications and associated conditions of GD I include splenectomy, persisting bone complications, pulmonary hypertension [3], Parkinson disease [4] and an increased risk of associated malignancies including multiple myeloma (MM) and hepatocellular carcinoma (HCC) [5,6].

Gaucher disease is the first disorder for which purified enzyme, administered intravenously, has shown to be effective in reversing most of the symptoms. Currently, three recombinant enzymes are available (imiglucerase, Cerezyme®, Genzyme Corporation, Cambridge, MA, USA; velaglucerase alfa, VPRIV®, Shire Human Genetic Therapies, MA, USA; and taliglucerase alfa, Protalix Biotherapeutics, Carmiel, Israel), of which imiglucerase was approved by the authorities already in the nineties while the latter two have only recently received approval (taliglucerase in the USA only). An alternative treatment option is substrate reduction therapy (SRT). The authorized compound is miglustat (Zavesca, Actelion Pharmaceutical, Switzerland). However, it is not the first choice treatment for GD I since it is only indicated for the treatment of mildly to moderately affected GD I patients for whom ERT is unsuitable.

Although imiglucerase is not authorized within the EU as an orphan drug, simply because the European orphan drug act was installed at a later stage, the questions with regard to long term outcome of this enzyme therapy and cost-effectiveness are not different compared to other orphan diseases, such as Fabry and Pompe disease [7]. In a recent paper we showed that long term enzyme replacement therapy for Gaucher disease can effectively reduce the incidence of splenectomy and bone complications, and will most likely result in a reduction in the risk of developing malignancies **(**van Dussen L, Biegstraaten M, Dijkgraaf MG, Hollak CE: Modelling Gaucher disease progression: long term enzyme replacement therapy reduces the incidence of splenectomy and bone complications**.***Submitted)*. The high cost of this treatment has been the subject of debates in the past and has set the benchmark for similar enzyme replacement therapies. A 2001 commentary by Clarke et al illustrates the debate on payment for ERT for GD in Ontario, Canada [8]. The increasing impacts on healthcare budgets of the growing number of orphan medicinal products have resulted in an increasing interest in health economic evaluations [9]. So far, for ERT in GD I, only limited cost-effectiveness and cost-utility analyses have been performed. Connock et al have reviewed the available data, primarily based on available literature as well as limited data from the Gaucher Registry [10]. Without the possibility for complex modeling, a comparison was made of cost-effectiveness of enzyme therapy versus no specific treatment for patients with GD I and a comparison of different dosage regimens. A literature review identified three studies that reported economic evaluations all of which produced very high incremental cost-effectiveness ratios even when high estimates of effectiveness were assumed. Even when the most generous assumptions about potential in cost savings are applied, the incremental cost effectiveness ratio (ICER) still exceeds £200,000 per quality adjusted life-year (QALY). However, it was acknowledged that no consideration was given to initiation of treatment in patients at different stages of disease, effect of immune reaction, adherence and drug holidays, comparative treatment effects and the potential for preventing more serious manifestations [10]. A more recent study from the UK Health Technology Assessment programme calculated that the minimal additional discounted QALYs needed for each year on ERT, in order for ERT to be judged as cost-effective, ranged between 4.2 and 4.8 for adults (assuming a willingness to pay per QALY of £30,000) [11].

In this paper, we modeled disease progression to assess the cost-effectiveness of ERT treatment for GD versus standard medical care without ERT in symptomatic patients from a societal perspective. The costs per year free of end-organ damage (YFEOD) were the primary outcome measure for the cost-effectiveness analysis. The costs per QALY were the primary outcome measure for the cost-utility analysis.

## Methods

This study was part of the TIPharma project T6-208: Sustainable Orphan Drug Development through Registries and Monitoring. Ethical approval was requested at the institutional review board, METC AMC. The institutional review board stated that ethical approval was not required.

### Markov model structure

A Markov state-transition model was built, in which different (consecutive) phases of Gaucher disease were distinguished. The model was built in DATA (Decision Analysis by Treeage) 3.5 and run in DATA Pro.

Eight mutually exclusive disease states were included: asymptomatic, signs/symptoms (any mention of signs/symptoms, organomegaly and/or cytopenia), recovery (recovery was only possible for patients in the signs/symptomatic disease state since bone complications and splenectomy are irreversible), splenectomy, bone complication (defined as one single bone complication), multiple complications (defined as multiple bone complications or a combination of bone complication (s), splenectomy, Parkinson disease or pulmonary hypertension), malignancy (defined as multiple myeloma/amyloidosis or hepatocellular carcinoma), and finally, the state of death.

Bone complications and malignancies were defined as distinctive complications/associated conditions because it was assumed that these disease manifestations had the most important impact on quality of life and health care and non-health care costs. Splenectomy was defined as a complication of GD I since it is performed in case of severe splenomegaly and/or cytopenia, and has been reported to be a risk factor for the occurrence of bone complications as well as certain GD I -associated malignancies. Further descriptive details of the disease states are reported in Table S1A of the Gaucher Model Appendix (provided as Additional file 1).

**Figure 1:**
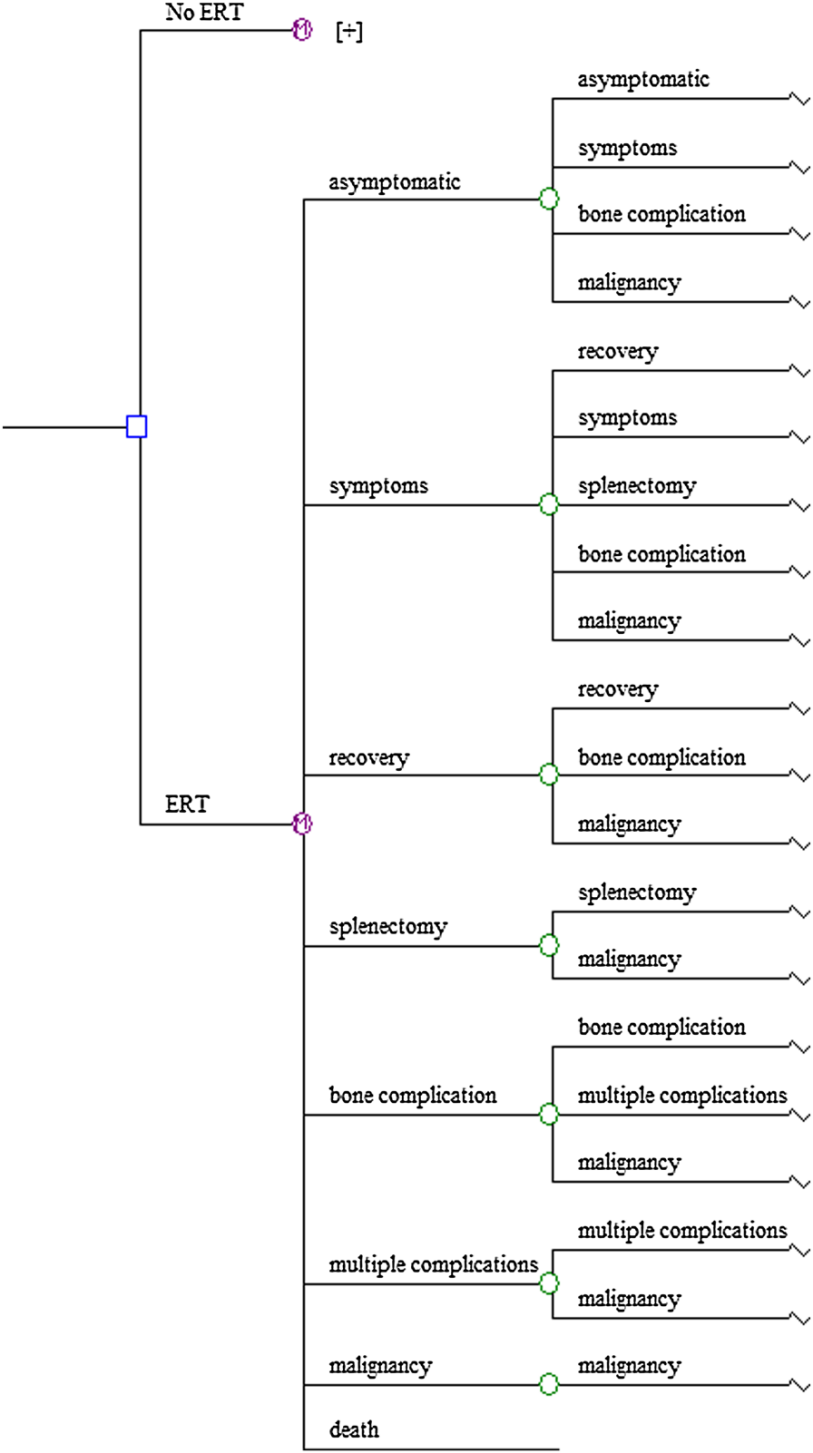
The Markov state-transition model of Gaucher disease.

The Markov state-transition model is designed to depict the prevalence and progression of disease signs/symptoms and complications in a simulated cohort of patients with GD I. During their lifetime patients progress through the successive disease states according to Figure 1. The cycle length of this Markov model represents 1year of life.

### Model data sources and assumptions

The model as proposed was developed with data from the Dutch Gaucher registry, validated with literature data and evaluated by expert opinion.

The Academic Medical Center is a national referral center for patients with GD I. This cohort consisted of all registered GD I patients in the Netherlands with a definite diagnosis of GD I based upon analysis of enzymatic activity and mutation analysis. Historical data were collected from all patients for whom a medical record before ERT was available (April 1991). For all patients who started ERT in the Netherlands after April 1991, data were collected prospectively up to September 1, 2011.

A natural history cohort and an enzyme replacement therapy (ERT) cohort were defined as discussed in a companion paper on long term enzyme replacement therapy for Gaucher disease **(**van Dussen L, Biegstraaten M, Dijkgraaf MG, Hollak CE: Modelling Gaucher disease progression: long term enzyme replacement therapy reduces the incidence of splenectomy and bone complications. *Submitted)*.

Considering data availability, the limited patient number, and the potential of unwelcome confounding by indication when contrasting the treatment and no treatment situations, we made several assumptions:

• state-transition probabilities for the natural course of GD I are only valid, when based on the period prior to the introduction of ERT therapy in the Netherlands;

• ERT allows for recovery from signs/symptoms; otherwise, it only affects the probability of progressing to the next disease state;

• health utilities for treated and untreated patients are similar as long as the patients are in the same disease state;

• health care volumes and related costs for treated and untreated patients are similar as long as they are in the same disease state, except for the costs of ERT medication.

### Transition probabilities

The time from one disease state to the next state was estimated by using Kaplan-Meier analysis and corrected for competing risks according to Ludbrook et al. [12]. It was estimated when 50% of the cohort in a certain disease state had reached the next state. Then, the yearly transition probability was calculated by dividing the cumulative proportion by the median time of follow-up, followed by a correction for the conditional nature of transition probabilities in the cyclic Markov model with a lifetime horizon (the Markov Correction factor [13]. If less than 50% of the cohort reached the next state, then time and cumulative proportion from the survival curve were used, where 4 patients were still at risk.

Transition probabilities were calculated for two different scenarios. In the base case, it was assumed that ERT will be offered to new patients as soon as they present themselves with signs/symptoms of GD I. Alternatively, we considered a historical perspective by taking the distribution of patients over the different disease states when ERT was introduced in the Netherlands in the year 1991 as the starting point. If Dutch GD I patients will continue to have access to ERT in the future, then the historical scenario will gradually convert into the base case^a^.

All yearly transition probabilities were assumed to be beta-distributed and are reported in Additional file 1: Tables S1B-1 (no ERT), S1B-2 (ERT, base case scenario) and S1B-3 (ERT, historical perspective) of the Additional file 1: Gaucher Model Appendix. Further details concerning the conceptualization and validation of the model structure and rates of disease progression are provided in the Additional file 1: Gaucher model Appendix as well.

### Mortality

For the mortality rate the background mortality in the healthy population was used based on the yearly survival rates (1-probability of survival) most recently published by Statistics Netherlands (Centraal Bureau voor de Statistiek: CBS.nl/en-GB/menu/home/default.htm, survival tables as of August 11, 2011), with the exception of the mortality rate for patients with a malignancy since the mortality rate is higher as compared to the background mortality.

### Health outcomes

Years free of end organ damage were calculated as the time in years spent in the asymptomatic, signs/symptomatic and recovery disease states.

Data on health status were obtained quarterly with the EQ-5D quality of life questionnaire. Previous research had determined the utility of each observed health score profile on the EQ-5D based on the time trade-off elicitation technique during interviews with adults from the UK general population [14]. Utilities range from minus 0.594, indicating serious health problems with mobility, self-care, usual activities, pain/discomfort, and mood, to unity, indicating no problems at all. By convention, death takes the value of zero. The resulting health utilities were averaged per patient per disease state - independently from therapy status -, and subsequently, per disease state over patients. Given the cycle length of one year in the Markov model, these mean health utilities equaled the number of QALYs generated during a single model cycle. Table 1 shows the mean health utilities per year along with their 95% confidence intervals after bias corrected and accelerated bootstrapping. Also reported in Table 1 are the mean yearly health utilities by disease state calculated with a Dutch equivalent of the UK-based health status scoring algorithm, which originated from a replication study by Lamers and colleagues [15]. The impact of applying the Dutch rather than the UK based general population preferences was addressed in a scenario analysis.

**Table 1:** Mean health utility by disease state

**Disease state**	N*	**Health utility**					
		**UK**	**95% LCL**	**95% UCL**	**NL**	**95% LCL**	**95% UCL**
Asymptomatic	**	0.93	0.8900	0.9700	0.93	0.8900	0.9700
Symptoms/recovery	17	0.8716	0.8177	0.9225	0.8897	0.8410	0.9349
Splenectomy	4	0.7532	0.6768	0.8215	0.7781	0.6990	0.8626
Bone complication	6	0.8614	0.7530	0.9685	0.8882	0.8027	0.9707
Multiple complications	13	0.7323	0.6601	0.8202	0.7981	0.7430	0.8638
Malignancy	1^#^	0.15			0.364		

*LCL*: lower limit of the confidence interval, *UCL*: upper limit of the confidence interval.

*Patients may contribute to more than one disease state.

**We used a foreign estimate of 0.93 (95% CI: 0.89-0.97) for healthy persons reported by Clarke et al. [16]. This estimate too was based on time trade-off based elicitation techniques.

^#^The observed estimate for the stage of malignancy was based on just 1 person, but well within the range of values expected for end-stage malignant disease (e.g. Brown et al. [17]).

### Health care volume and costs

Our approach to determine health care volume and costs mimics the approach in an earlier study on the cost-effectiveness for Fabry [7]. Health care costs included the direct and indirect medical costs of health care use as well as the indirect non-medical costs of production loss. Direct non-medical costs of private help, travel costs and non-reimbursed out-of-the-pocket expenses were not taken into consideration because of their expected relatively low impact. We assume that ERT would result in a reduction of these costs. Use and frequency of AMC care were collected from the 2004-2011 AMC hospital database based on the patient identification code. These data were linked to the available real unit costs from the AMC hospital ledger to arrive at the AMC costs [18]. Use and frequency of hospital care outside the AMC, out-of-hospital care and production loss in this patient population were derived from the quarterly disseminated Health and Labour questionnaires during the period May 2009 and April 2011. Subsequently these volumes were linked to unit costs from the Dutch costing manual 2010 for health care research [19]. The unit costs used were price-indexed for the year 2009 and are presented in Table 2.

**Table 2:** **Dutch unit costs** (€) **for resources used**

**Resource**	**Unit costs in 2009 euros**^#^	**Source**
Inpatient hospital day	596-1,036	AMC hospital ledger^##^
In-hospital day-care treatment	274 - 845	AMC hospital ledger
Enzyme replacement therapy per vial of 400 IU	1,985	Dutch College of health care insurance
Splenectomy	6,022	AMC hospital ledger
Other diagnostic and therapeutic procedures	Various	AMC hospital ledger
Outpatient hospital visit	90 - 460	AMC hospital ledger
Out-of-hospital visit		
General practitioner	28	Dutch costing manual
Physiotherapist	36	Dutch costing manual
Psychiatrist/psychologist^†^	91.5	Dutch costing manual
Occupational physician/other^††^	26	AMC hospital ledger
Social worker	65	Dutch costing manual
Alternative healer	60	Expert opinion^†††^
Productivity loss per hour^	30	Dutch costing manual

^#^In case of different base years the general price index figures from the Dutch costing manual 2010 have been used to derive 2009 estimates. ^##^Unit costs from the AMC hospital ledger for Gaucher patients include the costs of top referent health care. ^†^Weighted unit cost based on the assumption of 50%-50% distribution of visits over psychiatrists (€103) and psychologists (€80) respectively. ^††^Out-of-hospital visit to other care givers are assigned the lowest unit costs among the caregivers, i.e. the occupational physician. ^†††^The Nederlandse Mededingings Autoriteit prohibits the use of an advised tariff. Unit costs per consultation may vary considerably, depending on the type of alternative healer. As a proxy, the reported unit cost of 60 euro per visit is based on an indexed derivation of the advised 2000 tariff for an acupuncturist. ^Overall mean unit costs per hour, irrespective of gender, age, and occupation.

The mean yearly ERT costs per patient per disease state were determined by (i) averaging the number of vials of 400 IU per month per disease state (weighted by the lengths of dose-specific episodes during the disease state) for each patient, (ii) averaging these mean numbers of vials per month per disease state over patients, and (iii) multiplying this overall mean result by 12 and by the vial unit costs of ERT (see Table 2 for the latter). Table 3 shows the mean yearly ERT costs by disease state.

**Table 3:** Mean yearly volume and costs of enzyme replacement therapy by disease state

**Disease state**	N*	**Number of vials**	**95% LCL**	**95% UCL**	**Costs**	**95% UCL**	**95% UCL**
Signs/symptoms	28	76	62	91	151,147	123,501	180,088
Recovery	19	63	49	75	124,183	97,529	149,041
Splenectomy	6	78	50	111	155,082	98,443	220,004
Bone complication	9	102	75	132	202,348	149,319	261,573
Multiple complications	18	86	70	102	170,317	138,201	202,653
Malignancy	5	130	68	209	257,469	135,582	414,084

*LCL*: lower limit of the confidence interval, *UCL*: upper limit of the confidence interval.

*Patients may contribute to more than one disease state.

The costs of AMC hospital care were calculated by taking the product sum of hospital resources used and their respective unit costs. The costs were averaged per patient (both treated and untreated) per disease state per year of follow-up, and subsequently per disease state over patients to get the mean yearly AMC costs per disease stage (Table 4).

**Table 4:** Mean yearly numbers and costs of diagnostic and therapeutic procedures* **in the AMC by disease state**

Disease state**	**Mean costs per procedure in €**	**Number of procedures**	**95% LCL**	**95% UCL**	**Costs in €**	**95% LCL**	**95% UCL**
Asymptomatic (N = 4)	20.9	70.2	25.0	102.4	1,470	455	2,152
Signs/symptoms (N = 25)	33.5	86.1	63.5	110.5	2,887	1,974	3,885
Recovery (N = 20)	32.4	94.3	79.6	110.1	3,055	1,708	4,858
Splenectomy (N = 5)	35.4	136.6	106.8	171.1	4,836	2,544	7,145
Bone complication (N = 8	40.9	106.0	73.9	154.9	4,337	1,590	9,313
Multiple complications (N = 22)	24.0	91.3	75.2	109.8	2,194	1,652	2,826
Malignancy (N = 4)	76.4	360.1	68.0	646.3	27,523	4,786	51,722

*LCL*: lower limit of the confidence interval, *UCL*: upper limit of the confidence interval.

*AMC inpatient days too were counted as procedures here.

**Patients may contribute to more than one disease state.

The volume and costs of all other used health care resources as derived 3-monthly from the Health and Labour questionnaires were averaged per patient per disease state independently from treatment status, multiplied by 4 to arrive at yearly mean estimates per patient per disease state, and then averaged per disease state over patients. Table 5 shows the mean yearly volumes and costs of out-of-hospital consultations by disease state, of which the mean total values are included in the Markov model.

**Table 5:** **Mean yearly numbers and costs of out**-**of**-**hospital consultations**^§^**by disease state**

Disease state*	**Volumes**	**95% LCL**	**95% UCL**	**Costs in €**	**95% LCL**	**95% UCL**
(No) signs/symptoms (N = 10)**	3.6	1.1	7.8	121	31	272
Recovery (N = 9)	16.8	6.3	28.3	641	229	1,097
Splenectomy (N = 4)	6.0	0.3	11.6	299	8	589
Bone complication (N = 6)	12.7	1.6	26.4	449	51	939
Multiple complications (N = 13)	6.8	2.9	11.2	245	97	421
Malignancy (N = 1)	2			56		

*LCL*: lower limit of the confidence interval, *UCL*: upper limit of the confidence interval.

*Patients may contribute to more than one disease state. **Data from two asymptomatic patients have been added to data from eight symptomatic patients to improve precision; the estimates were applied to both, the asymptomatic as well as symptomatic Markov disease state. § Patients in the asymptomatic and symptomatic disease states reported visiting the general physician, physiotherapist, company physician, and alternative healer. Patients in recovery reported visiting the general physician, physiotherapist, social worker, and alternative healer. Patients with a splenectomy reported visiting the general physician, social worker, and alternative healer. Patients with a bone complication reported visiting the general physician, physiotherapist, and company physician. Patients with multiple complications reported visiting the general physician, physiotherapist, psychologist/psychiatrist, company physician, social worker, and alternative healer. The patient with a malignancy reported visiting the general physician.

For 70.5 person-years of questionnaire data on health care use in hospitals other than the AMC as tertiary referral centre only 33 inpatient hospital days were reported, equivalent to about €15,000 in total. For the model these costs were considered trivial. Likewise the total costs of self-reported, prescribed medications other than ERT during these person-years - €4,433 - seemed redundant and have been discarded as well.

Table 6 shows the mean yearly costs of production loss resulting from sick leave and (permanent) work disability as a consequence of GD I. The costs for production loss follow the human capital costing approach and were derived in successive calculation steps. For each patient with paid work the mean number of working hours per day and the mean number of working hours per week were derived from the Health and Labour questionnaire. The mean volume of sick leave in number of days per 2-week period was calculated per patient per disease state. Treated and untreated patients were taken together. This volume was then multiplied by 26 and by the overall mean number of working hours per day to arrive at the yearly mean production loss per patient with paid work per disease state. For patients with (permanent) work disability because of GD I the estimated yearly volume of production loss was based on the overall mean number of working hours per working day and overall mean number of working days per week for patients with paid work. For patients who did not have paid work for reasons other than GD I a zero volume of production loss was assumed.

**Table 6:** Mean yearly indirect costs of production loss by disease state

**Disease state**	N*	Costs of production loss in €**	**95% LCL**	**95% UCL**
Signs/symptoms	9	0	0	0
Recovery	9	0	0	0
Splenectomy	4	13,698	0	27,396
Bone complication	6	10,002	0	20,004
Multiple complications	13	10,615	0	21,230
Malignancy	1	73,057		

*LCL*: lower limit of the confidence interval, *UCL*: upper limit of the confidence interval.

*Patients may contribute to more than one disease state.

**Based on the human capital valuation method. Volume data in number of hours can be derived by dividing the cost figures by the unit cost per lost working hour (or €30).

The calculated or defined yearly volume of production loss in hours per patient per disease state was multiplied by the average unit cost per hour of production loss irrespective of the gender or age of the patient (€30, see Table 2). Subsequently these costs were averaged per disease state over patients.

### Analysis

The model was run from a lifetime perspective, starting asymptomatically at birth until the age of 85 years or until death. Hypothetical cohorts of treated and untreated patients in the Markov model were compared for the primary outcome parameters of the cost-effectiveness and cost-utility analyses by dividing the lifetime costs difference by the difference in lifetime years free of end-organ damage or by the difference in lifetime QALYs respectively.

In the *base case scenario*, patients entered the model at birth; ERT was initiated when signs/symptoms developed; the costs only included the medical costs; no discounting of effects or costs was performed. Sensitivity analyses have been performed concerning the choice of discount rate (discounting of effects by 1.5% and costs by 4% instead of no discounting for both) [20] and for the Dutch instead of the UK time trade-off based health utility algorithm. Considering that we assumed equal health utilities and costs (excluding ERT medication) for treated and untreated patients during the same disease states, we put most emphasis on assessing the impact of parameter uncertainty resulting from the transition probabilities from one disease state to the next. To this end, a Monte Carlo simulation was performed with 1,000 second-order draws from the beta-distributed yearly transition probabilities in the Markov model to represent parameter uncertainty with each single draw including 100 first-order trials to represent patient heterogeneity. Additionally, the impact of parameter uncertainty concerning the health utility and costs estimates were assessed assuming triangular distributions (with mean estimates taken as the most likely values and the 95% confidence limits taken as the minimum and maximum values).

The main results for the incremental costs and QALYs gained by ERT versus standard medical care are reported as a cost-effectiveness plane and as a cost-effectiveness acceptability curve for willingness to pay (WTP) values up to 10,000,000 euro per QALY.

### Scenario analyses

Following the base case scenario we ran three alternative scenarios:

– (a) adding the costs of production loss to the medical costs, assuming a maximum productive period of 40 years (between one’s 25^th^ and 64^th^ years of age).

– (b) reducing the costs of ERT by 25% to reflect the actual (minus 15%) and perhaps potential (minus another 10%) variation in costs per vial

– (c) taking the historical perspective by accounting for the time that ERT first became available for the Dutch market.

## Results

### Health outcomes, medical costs, incremental cost-effectiveness ratio, and cost-effectiveness acceptability

**Figure 2:**
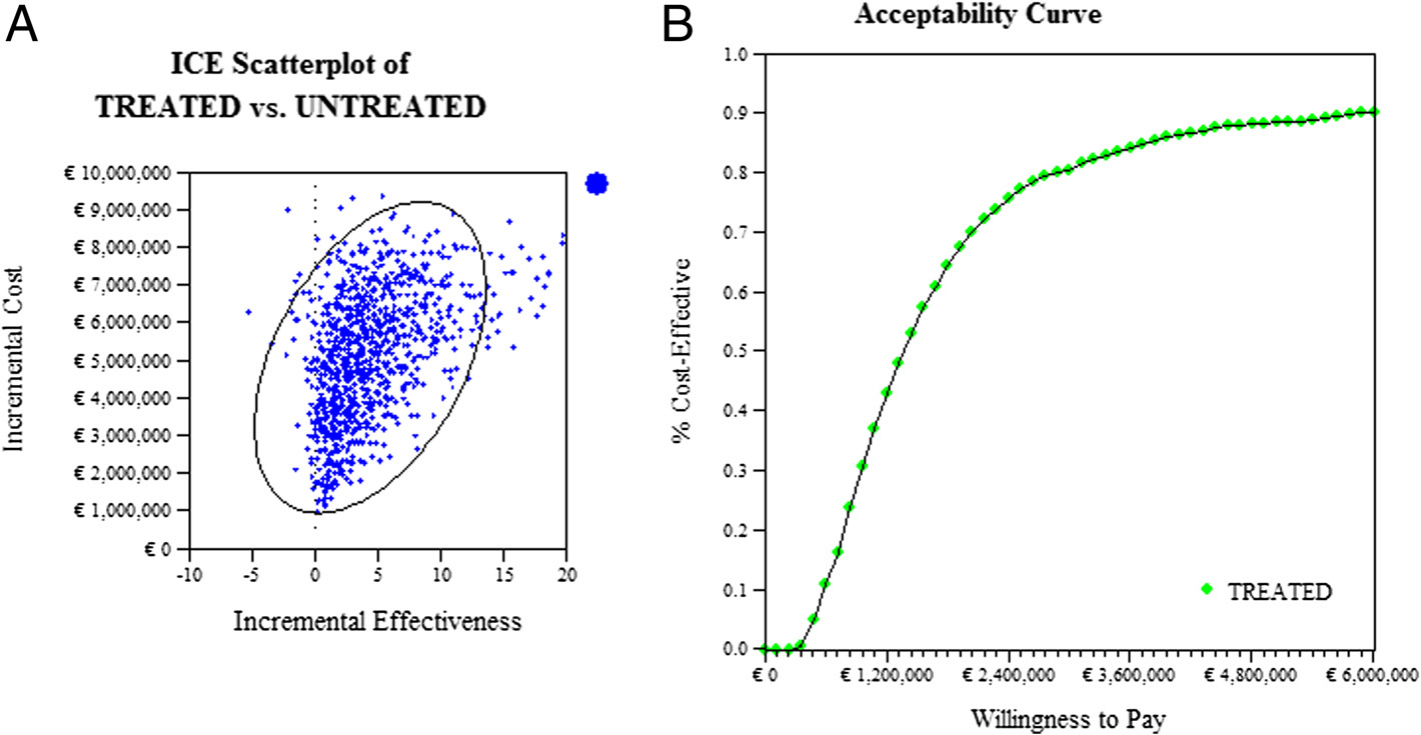
**Enzyme replacement therapy against standard medical therapy after Monte Carlo simulation of transition probabilities. (A)** Scatterplot of undiscounted incremental medical costs and QALYs **(B)** corresponding cost-effectiveness acceptability curve.

**Figure 3:**
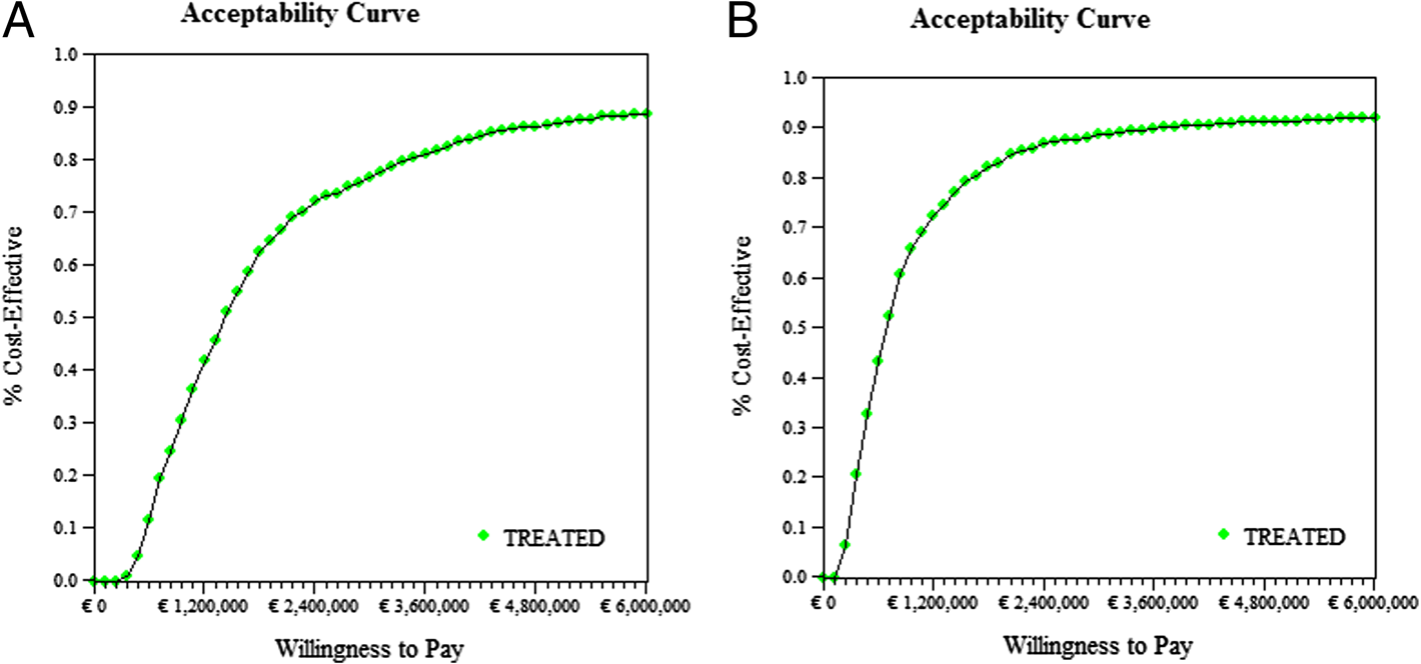
**Cost-effectiveness acceptability curves for enzyme replacement therapy against standard medical care after Monte Carlo simulation of all model parameters. (A)** Undiscounted **(B)** discounted.

Under the base case scenario, GD I patients receiving standard medical care generated 48.9 YFEOD and 55.86 QALYs during their lifetime. If treated with ERT, these numbers increased to 61.7 YFEOD and 62.13 QALYs, resulting in health gains of 12.8 YFEOD and 6.27 QALYs respectively. Not treating Gaucher patients with ERT resulted, on average, in €171,780 over a patient’s lifetime. Treatment with ERT resulted in a mean lifetime costs of €5,716,473, a difference of €5,544,693. The incremental cost-effectiveness ratios of ERT treatment against standard medical care only equaled €434,416 per YFEOD and €884,994 per QALY (Tables 7 and 8). Figure 2a shows the incremental medical costs on the Y-axis against the incremental QALYs on the X-axis of ERT treatment against standard medical care after Monte Carlo simulation of the transition probabilities in the Markov model. Most simulations end up in the upper right quadrant (96.3%), indicating increasing costs and QALYs. Figure 2b shows the corresponding cost-effectiveness acceptability curve with the probability of ERT being cost-effective on the Y-axis for different WTP values on the X-axis. The probability of ERT treatment being cost-effective equaled 0.25, 0.5 and 0.75 at WTP values of about €860,000, €1,370,000, €2,360,000 per QALY respectively. These latter results changed into about €850,000, €1,420,000, €2,810,000 per QALY respectively when taking uncertainties regarding health utilities and costs into account as well (Figure 3a).

**Table 7:** **Incremental costs per year free of end organ damage gained under different scenarios**, **undiscounted** (**upper rows**) **and *discounted*** (***lower rows***)

**Scenario**	**YFEOD**			**Costs**			**ICER**
	**ERT**	**no ERT**	**Δ**	**ERT**	**no ERT**	**Δ**	
Base case	61.70	48.90	12.80	€5,716,473	€171,780	€5,544,693	€434,416
	*37.77*	*31.97*	*5.80*	€*1*,*206*,*933*	€*50*,*048*	€*1*,*156*,*885*	€*199*,*559*
Production loss included	61.70	48.90	12.80	€5,772,897	€294,226	€5,478,670	€429,243
	*37.77*	*31.97*	*5.80*	€*1*,*216*,*954*	€*71*,*956*	€*1*,*144*,*998*	€*197*,*508*
25% ERT costs reduction	61.70	48.90	12.80	€4,338,430	€171,780	€4,166,649	€326,449
	*37.77*	*31.97*	*5.80*	€*918*,*801*	€*50*,*048*	€*868*,*753*	€*149*,*857*
Historical I*	19.72	17.90	1.82	€5,202,872	€109,342	€5,093,530	€2,803,382
	*14.83*	*13.70*	*1.14*	€*2*,*391*,*020*	€*54*,*143*	€*2*,*336*,*877*	€*2*,*057*,*891*
Historical II*	12.10	10.14	1.96	€6,198,258	€112,774	€6,085,484	€3,111,478
	*9.27*	*8.01*	*1.26*	€*3*,*031*,*062*	€*59*,*253*	€*2*,*971*,*809*	€*2*,*363*,*665*

*YFEOD*: years free of end organ damage, *ICER*: incremental cost-effectiveness ratio. *The historical scenarios were based on the patient distribution in the Netherlands when ERT was introduced in April 1991 and were derived from medical histories of patients referred to the AMC until September 2011. The first historical scenario includes the asymptomatic stage, the second historical scenario excludes the asymptomatic stage. While running the historical scenarios, two model adjustments were made: 1) the probability of death was adjusted for the mean age of 36 (Historical I) or 38 (Historical II) years for patients in the cohort at the time of ERT market introduction in the Netherlands; 2) the time horizon too was adjusted for the mean age of the cohort in 1991 and became 49 (Historical I) or 47 (Historical II) years instead of 85.

€: amount of money in euros.

Δ: difference between between the scenarios with versus without ERT.

**Table 8:** *Incremental costs per quality adjusted life year gained under different scenarios, **undiscounted** (**upper rows**) **and *discounted*** (***lower rows***)

**Scenario**	**QALY**			**Costs**			**ICER**
	**ERT**	**no ERT**	**Δ**	**ERT**	**no ERT**	**Δ**	
Base case	62.13	55.86	6.27	€5,716,473	€171,780	€5,544,693	€884,994
	*37.33*	*34.65*	*2.67*	€*1*,*206*,*933*	€*50*,*048*	€*1*,*156*,*885*	€*432*,*540*
Production loss included	62.13	55.86	6.27	€5,772,897	€294,226	€5,478,670	€874,456
	*37.33*	*34.65*	*2.67*	€*1*,*216*,*954*	€*71*,*956*	€*1*,*144*,*998*	€*428*,*096*
25% ERT costs reduction	62.13	55.86	6.27	€4,338,430	€171,780	€4,166,649	€665,043
	*37.33*	*34.65*	*2.67*	€*918*,*801*	€*50*,*048*	€*868*,*753*	€*324*,*812*
Historical I	33.00	28.50	4.50	€5,202,872	€109,342	€5,093,530	€1,131,036
	*24.27*	*21.35*	*2.92*	€*2*,*391*,*020*	€*54*,*143*	€*2*,*336*,*877*	€*800*,*359*
Historical II	30.28	24.65	5.64	€6,198,258	€112,774	€6,085,484	€1,079,504
	*22.52*	*18.78*	*3.74*	€*3*,*031*,*062*	€*59*,*253*	€*2*,*971*,*809*	€*794,656*

*QALY*: quality adjusted life year, *ICER*: incremental cost-effectiveness ratio.

*For explanation of Historical I and Historical II, see notes Table 7.

€: amount of money in euros.

Δ: difference between between the scenarios with versus without ERT.

### Discounting effects and costs and applying dutch general population preferences for health states

After discounting of effects by 1.5% yearly, GD I patients receiving standard medical care generated 31.97 YFEOD and 34.65 QALYs during their lifetime, while patients on ERT generated 37.8 YFEOD and 37.33 QALYs. After discounting of costs by 4% yearly, Gaucher patients not on ERT treatment generated €50,048, while ERT patients generated €1,206,933 during their lifetime. Discounting decreased the incremental cost-effectiveness ratio from €434,416 to €199,559 per YFEOD gained and from €884,994 to €432,540 per QALY gained. Figure 3b shows the cost-effectiveness acceptability of ERT against standard medical care following discounting, after Monte Carlo simulation of all model parameters and for different willingness to pay values per QALY. The probability of ERT treatment being cost-effective equaled 0.25, 0.5 and 0.75 at WTP values of about €400,000, €690,000, €1,340,000 per QALY respectively.

Applying the Dutch general population preferences for health status rather than the ones from the UK showed slightly higher incremental costs per QALY ratios from €884,994 per QALY to €918,709 per QALY undiscounted and €432,540 per QALY to €451,647 per QALY discounted.

### Scenario analyses

The undiscounted and discounted results from the scenario analyses with YFEOD and QALYs as the primary clinical outcomes are reported in Tables 7 and 8 respectively.

The Tables show that including the costs of production loss resulted in marginally lower incremental costs per YFEOD (minus €5,173 undiscounted; minus €2,051 discounted) and incremental costs per QALY (minus €10,538 undiscounted; minus €4,444discounted) in comparison with the base case scenarios. Reduction of the costs of ERT substantially decreased the undiscounted incremental costs per YFEOD from €434,416 to €326,449 (discounted from €199,559 to €149,857) and the undiscounted incremental costs per QALY from €884,994 to €665,043 (discounted from €432,540 to €324,812).

Both variants of the historical scenario were accompanied by substantial increases of all the incremental cost-effectiveness ratios.

## Discussion

### Summary and significance of major findings

The modeling of ERT for GD I with a lifetime time horizon of 85 years demonstrated important gains in effectiveness with 12.8 extra years free of end-organ damage (discounted: 5.8) and 6.27 additional QALYs (discounted: 2.67). With average yearly ERT medication costs easily ranging between €124,000 and €258,000 from the moment signs/symptoms develop, the extra lifetime costs of ERT compared with standard medical care without ERT amounted to €5,544,693 per patient on average (discounted: €1,156,885), resulting in incremental cost-effectiveness ratios of €434,416 per YFEOD (discounted: €199,559) and €884,994 per QALY (discounted: €432,540). The acceptability of ERT for GD I may top a high 96% from a health economic perspective, but this obviously will depend on society’s willingness to pay per QALY. Given current standards for affordable health care interventions, these results may leave the treatment of GD I with enzyme replacement therapy under continuing debate. However, there are some study limitations (see below) that forced us to take a conservative stand while quantifying model parameters. This, in turn, necessarily qualifies the major findings as conservative ones.

The societal perspective of this economic evaluation is dominated by the health care costs. Adding the costs of production loss based on a human capital approach, i.e. taking all current and future production loss resulting from sick leave and work disability into account, only marginally changed the major findings for the better (lower incremental cost-effectiveness ratios). The application of the (nationally advised) friction cost method to the costing of production loss - resulting from sick leave and less productivity while at work - would have resulted in even smaller changes and has justifiably not received further attention here.

Both historical scenarios reflecting relatively delayed starts of ERT when patients in a population already are in various stages of the disease at the time of its market introduction showed worse off incremental cost-effectiveness ratios compared to the base case scenario. This indicates that starting ERT right when patients are in need of it in an early signs/symptomatic stage, is probably the most sensible approach, both from a medical as well as health economic perspective.

One should further note that the significance of ERT provision for the national health care budget is not just a matter of costs per QALY gained, but also a matter of demand for health care, which is low here given that Gaucher disease is rare. Whether society is willing to pay more for treatments of rare disorders than for more prevalent diseases is uncertain. This discussion can only be started once cost-effectiveness analyses are carried out and costs per QALY are known.

### Study limitations

Important limitations of this ERT evaluation study include the small size of the studied cohort and the non-randomized, partly retrospective study design. Simplifications of the model structure that reflects the course of the Gaucher disease and making assumptions during data analysis and estimation of model parameters were needed to exert as much control as possible over factors other the enzyme replacement therapy itself that may explain differences in model outcomes between treated and untreated patient cohorts.

To contain possible distortions due to ‘confounding by indication’ and obtain an adequate description of the natural course of Gaucher disease when most Gaucher patients actually already receive ERT, we obtained all data on the natural course of disease progression retrospectively from patient records before the era of ERT. However, data on signs/symptoms and complications might not have been recorded as rigorously as would have been the case in a prospectively designed study. We therefore cannot exclude the possibility that the rate of disease progression in untreated patients has been underestimated in the present analysis.

At the same time, transition probabilities for disease progression among patients receiving ERT have probably been overestimated, because an accurate set of patients starting on treatment in an early signs/symptomatic stage could not be identified without a considerable risk of selection bias by overrepresentation of patients who progress slowly. The reported base case scenario approximates the ideal scenario for upcoming Gaucher cases, because the transition probabilities used in that scenario partly reflect delayed start of therapy in various disease stages. As a result, we have perhaps underestimated the full potential of ERT.

Another limitation of our study concerns the worldwide imiglucerase shortage. Questionnaires to assess quality of life were distributed from June 2009 onwards which coincides with the period of shortage. One might argue that the shortage could have had an impact on the quality of life of patients. However, based on the literature discussing the impact of the shortage on patients’ well being [21,22], this effect is assumed to be small.

Finally, ERT dose and frequency of ERT administration differ between countries, which may impact transferability of these results to other countries. ERT regimen will influence costs of treatment and may influence disease course. Nonetheless, a comparison between AMC data and literature data, details of which are reported in the appendix, revealed that data used in our model of disease course are in line with results reported in the literature.

### Implications and recommendations for future studies

Although this study on Gaucher disease shows an 7-fold more favorable cost-effectiveness ratio when compared to a recently accomplished similar analysis for Fabry disease [7], costs per QALY are well above what is generally perceived as affordable. In case of Gaucher disease, not meeting the cost-effectiveness limits (for non-rare diseases) is clearly not resulting from a lack of effectiveness of the ERT with almost 13 years free of end-organ damage for each individual patient. We plea for lowering the unit costs of ERT and making these unit costs negotiable in public-private partnerships that incorporate some risk coverage for pharmaceutical companies with highly promising, ‘priceless’ drugs for rare diseases in their portfolio. Enzyme replacement therapy for Gaucher disease seems one of them.

In addition, the outcomes of the current study can fuel the debate on drug pricing as we show that price drops of ERT may substantially improve the incremental costs per QALY ratio. In addition, as ERT for GD I is widely implemented and reimbursement guaranteed in most Western countries during more than 20 years, the current analysis may also serve as a benchmark for discussions on orphan drug price limits. We suggest that the excellent effectiveness and safety of ERT has contributed importantly to this situation.

An important lesson is to be learned from the limitations cited for our study. Sufficient and reliable data on the natural course of a disease are essential not only to assess the effectiveness of future treatments, but also from a cost-effectiveness perspective. It has become clear that studies are mostly restricted to uncontrolled trials or observational studies after a certain drug has become registered. This is specifically the case when registration took place under ‘exceptional circumstances’, meaning that additional clinical trial data will probably not become available during the post-marketing authorization period. Therefore we argue for the systematic collection of natural history data, within a disease registry rather than a company sponsored drug registry [23], aiming to obtain the relevant data that are needed to perform adequate efficacy but also cost-effectiveness analyses. Otherwise, such analyses will be permanently hampered by shortcomings as reported in this paper.

## Endnote

^a^Please note that the base case scenario relates to scenario B in the companion paper on Long term enzyme replacement therapy for Gaucher disease, while the historical scenario relates to scenario A in that paper.

## Competing interest

The submitted work was supported only by Top Institute Pharma; CH has received reimbursement of expenses and honoraria for lectures on the management of from Genzyme, Actelion, Protalix and Shire HGT. MB has received reimbursement of expenses and honoraria for lectures on the management of from Genzyme, Actelion and Shire HGT. MB and CH donated the honoraria to the Gaucher Stichting, a foundation that supports research in the field of lysosomal storage disorders. LvD has received travel reimbursements at two occasions (Genzyme, Protalix). MD has no relationships with companies that might have an interest in the submitted work in the previous three years; none of the authors has non-financial interests that may be relevant to the submitted work.

## Author contribution

LD and MD developed the cost-effectiveness model. CH and MB contributed to model development and data collection. LD, MB and MD analysed the data. LD and MD wrote the manuscript. MB, CH critically revised the manuscript. All authors read and approved the final manuscript.

## Supplementary Material

Additional file 1 Gaucher Model Appendix. Click here for file
