# Supplementary material for: Cost-effectiveness of enzyme replacement therapy for type 1 Gaucher disease
Source: Orphanet J Rare Dis. 2014 Apr 14;9:51. doi: 10.1186/1750-1172-9-51 (PMC4022049; doi:10.1186/1750-1172-9-51)
Supplement: Additional file 1 — Gaucher Model Appendix. [file 1750-1172-9-51-S1.doc]

**Gaucher Model Appendix: Conceptualization and validation of the model structure and rates of disease progression**

*Transition probabilities for untreated patients*

Enzyme replacement therapy became available in the Netherlands in April 1991. Historical data on the progression of disease were retrieved from clinical records in all 90 patients. For the assessment of the time to onset of signs/symptoms the entire AMC cohort was analyzed, since only signs/symptomatic patients are treated. For the assessment of each consecutive disease stage only patients that had reached a particular disease stage before the era of ERT were included in the analysis. The reason for this is that inclusion of untreated patients after ERT became available would lead to an underestimation of disease progression as a result of bias by indication, as these patients exhibit milder disease manifestations. Thus, patients who had reached a particular disease stage prior to April 1991 were included in the analysis of the natural course of progression from that particular disease stage to the next, e.g. from signs/symptoms to bone complications. If a patient showed no progression before April 1991, then April 1991 was noted as the end of follow-up and the case was censored .

*Transition probabilities in the base case scenario*

Transition probabilities were calculated for two different scenarios. In the base case scenario, it was assumed that ERT will be offered to new patients as soon as they present themselves with signs/symptoms of Gaucher disease. The number of patients that was diagnosed and referred without any delay to the Academic Medical Center since 1991 was insufficient to reliably estimate progression rates from one disease state to the other.

To estimate the true transition probabilities as precisely as possible while making optimal use of the clinical data available, the entire cohort was included. The time of onset of a complication was calculated from the time that a patient entered the disease state independently from treatment status at that time. This implies that in most cases ERT was started at some time in the course of a disease state instead of at the start of that disease state period. Consequently, transition probabilities were probably overestimated since patients in a particular disease state were only partially treated with ERT.

Two exceptions were made:

The first exception was made for the transition from signs/symptoms to recovery. Applying the base case scenario would mean that a patient who recovers 6 months after the start of treatment would for example recover after 10 years if the time to recovery was calculated from the start of signs/symptoms. This would underestimate the effect of ERT since a patient is only expected to recover after initiation of treatment. The probability for the transition from signs/symptoms to recovery was therefore calculated from the time that a patient started treatment.

The second exception was made for the transition from recovery to bone complications. A patient who started treatment twenty years after the first signs/symptoms appeared may show residual disease resulting in an increased risk of developing bone complications. This risk might not apply to patients who started treatment only a few months after the appearance of the first signs/symptoms. To correct for this, we calculated for all patients the time from signs/symptoms to the start of ERT. This duration was added to the duration from recovery to bone complication or end of follow-up in case of censoring, effectively lengthening the time to complications after recovery and thus lowering the transition probability.

*Transition probabilities in the historical scenario*

Alternatively, we considered a historical perspective by taking the distribution of patients over the different disease states when ERT was introduced in the Netherlands in the year 1991 as the starting point. When using the entire Dutch cohort this would imply a distribution as shown in the left column of Table S1C. The high percentage of asymptomatic patients follows from the lifetime perspective of the model; patients were in the asymptomatic phase from birth to the development of signs/symptoms, and, consequently, all patients who developed signs/symptoms after 1991 are in the asymptomatic phase in 1991. Since one-third of the Gaucher population at an outpatient clinic being asymptomatic does not reflect the true situation, an alternative distribution was based on the assumption that only patients with signs/symptoms and / or complications are normally visiting the outpatient clinic, effectively resulting in zero patients in the asymptomatic phase and higher percentages of patients in the remaining phases (see right column of Table S1C).

Patients in this scenario include those who already had a history of longstanding Gaucher disease to mildly affected patients as well as recently diagnosed patients with still limited disease manifestations. In this scenario, the time of onset of a complication was calculated from the time that a patient started treatment, implying that complications may arise as a result of irreversible disease. For example, in a patient with extensive bone disease, a bone crisis may occur 6 months after start of ERT. Therefore, transition probabilities for this scenario are generally higher than those in the base case scenario.

*Assumptions*

As discussed in our recent paper, GD associated malignancies occurred almost exclusively in the ERT treated patients. However, associated malignancies are certainly not a consequence of treatment as evidenced by numerous studies and case reports describing the occurrence of these malignancies in untreated patients (see for example Lee et al 1982 ). We showed that ERT effectively prevents / reduces progression to more advanced stages of disease, suggesting that ERT is able to reduce the frequency of Gaucher associated malignancies. This is supported by the observations that: 1) hepatocellular carcinoma in GD I patients is exclusively seen in splenectomized patients and splenectomies were only performed in untreated patients, 2) only one patient developed an MGUS while treated. MGUS in itself is not a malignancy, but the yearly risk of transition from an MGUS to a multiple myeloma or other lymphatic malignancy is 1% .

Therefore we decided to use the transition probabilities calculated for the ERT cohort (base case scenario) for the chance of developing a malignancy as transition probabilities in the natural history cohort. Since these transition probabilities were calculated based on a period including treatment, they might underestimate the actual probability of developing a malignancy. The chance of developing a malignancy while treated was assumed to be smaller, but not 0 since GD can present itself with an MGUS without signs/symptoms of GD being present and progression from MGUS to MM despite ERT has been known to occur . We decided to use very low transition probabilities (near zero) thereby reflecting the more likely probabilities of developing a malignancy in the ERT group.

Secondly, deriving time and cumulative proportion to calculate the transition probability from splenectomy to malignancy when 4 patients were still at risk would result in a transition probability of 0 since the malignancy occurred at a later stage. Since this would underestimate the chance of developing a malignancy after a splenectomy, we decided to incorporate a more realistic transition probability derived when 2 patients were still at risk.

Finally, no patients in the Dutch Gaucher cohort progressed to the phase splenectomy while treated. However, there are anecdotal reports to suggest that ERT might not be able to prevent the need for a splenectomy in rare circumstances such as the development of neutralizing antibodies to ERT or extensive fibrosis rendering a patient irresponsive to treatment. The decision was made to incorporate this course in the Markov model, albeit with a very low transition probability.

*Data source validation*

Numerous studies both on the natural history of GD and the effect of ERT on disease progression have been published. However, the fact that our data have been prepared for estimation of specific parameters in a Markov state-transition model hampers a direct comparison to the data cited in the literature. Most studies, for instance, report the percentage of patients in a cohort that have experienced a bone complication, but do not distinguish between a first and second complication or between splenectomized and non-splenectomized patients. In order to validate the data we used to construct our model, our data are presented in such a way that they can be compared to the data presented in the literature.

Development of signs/symptoms

Age at diagnosis and severity of signs/symptoms is dependent on the population studied. Especially, Ashkenazi Jewish populations have a relatively high proportion of N370S homozygotes who present with less severe disease.

Table S1D-1 presents the mean age at diagnosis for the Dutch cohort as compared to the patients from the ICGG and the study by Zimran and Giraldo divided by genotype. It can be concluded that the Dutch cohort represents a general non-Ashkenazi Jewish population.

Table S1D-2A offers an overview of studies in cohorts with a comparable genetic make-up. The populations that most closely resemble the Dutch cohort are those described by Giraldo and Drugan . Papers by Reich , Lee , Rodrigue and Revest were included for comparison, but do not specify the genetic / ethnic background of their population. Mean age at diagnosis is the Spanish cohort described by Giraldo et al was 24 years . This study is not included in the table since it consists of both treated and untreated patients. However, since treatment can only be started after a diagnosis is made, this would not influence age at diagnosis.

Comparison to AMC data

Mean age at signs/symptoms in our cohort (N=70) was 25.7 years. Our data are in line with data reported by Revest and Giraldo, but studies by Rodrigue and the Registry report a lower age at diagnosis . Rodrigue’s cohort consists of patients evaluated for the first ERT trials. This might be a selection of more severely affected patients, explaining the younger age at diagnosis. It is difficult to interpret data from the Registry. This cohort consists of a relatively high percentage of N370S homozygous patients (23% versus 9% in the Dutch cohort) in whom one might expect a higher age at diagnosis. On the other hand, type II and III patients are included in the Registry. Also, physician awareness and family-screening are more common among Ashkenazi Jewish patients who are well represented in the Registry.

Of note is that some studies cite an age at diagnosis, while other studies cite the age at first signs/symptoms. Differences between these two might arise in case of diagnostic delay or if a diagnosis is made as a result of family-screening when signs/symptoms might not necessarily be present.

Natural history data on developing complications: bone complications, Sx

Table S1D-2A offers an overview of studies in cohorts of comparable genetic make-up.

Comparison to AMC data

Twenty-four of 73 patients in our natural history cohort had undergone a splenectomy < April 1991 (33%), while 22 of 73 (30%) patients experienced at least one bone complication. Our data on the percentage of splenectomized patients are higher than the study by Reich, in line with Registry data, but lower as compared to studies by Lee and Drugan .

The definition of a Gaucher disease related bone complication is variable. Some studies cite ‘atypical’ bone pain as such, while others limit their reports to more readily objective bone complications such as avascular necrosis or pathological fractures. This is reflected by the variable percentages of patients with bone involvement reported ranging from 15% of Registry patients with a history of fractures to 69% of patients in Rodrigue’s study reporting on bone pain. In our study bone complications are defined by bone crises, osteonecrosis and pathological fractures, while atypical bone pain was not included. The 30% of bone complications that we found in our cohort is within the range reported in the literature.

ERT data: recovery

Numerous studies have reported on the response of haemoglobin level, platelet count and / or organomegaly. We have limited our comparison to those studies that enabled us to evaluate the individual response of patients included. If possible the criteria used in our paper were applied to the studies cited. Table S1D-2B offers an overview of these studies. A study that is not incorporated in the table, but should be mentioned is an analysis from the ICGG performed in 2008 . This paper offers a benchmark analysis of the achievement of so-called ‘therapeutic goals’. Therapeutic goals were defined as described by Pastores et al . Forty-one percent of all patients had achieved all 6 therapeutic goals after 4 years of treatment.

Comparison to AMC data

Eighteen out of 28 signs/symptomatic patients (historical perspective) recover (64%). Fifty percent of the patients recovered within 3.6 years from the start of treatment.

The criteria we used to define ‘recovery’ differ from the therapeutic goals analyzed in the Registry paper (2008) as illustrated in table S1E-1 . The Registry study offers results after 4 years of ERT. Within the AMC cohort, ~50% had recovered from signs/symptoms after approximately 4 years of ERT, which is comparable to the percentage of patients achieving 6 of 6 treatment goals.

To enable a comparison with the studies mentioned in table S1D-2B, we re-analyzed the response to treatment of our patients for each of the 5 parameters separately (including the patients that showed progression to the phase ‘bone complication’):

Of the 28 signs/symptomatic patients:

-17 patients were anemic at the start of treatment.16 recovered (94%). Median time to recovery in 16 patients was 1.25 years (range 0.10-7.92 years).

-22 patients had a platelet count <100,000/uL at the start of treatment. In 16 patients the platelet count increased to values >100,000/uL during follow-up (73%). Median time to recovery in 16 patients was 1.67 years (range 0.12-14.55 years);

-19 patients had a liver volume >1.25 MN at the start of treatment. In 15 patients liver volume declined to values <1.25 MN (79%). Median time to recovery in 15 patients was 2.5 years (range 0.42-7.01 years);

-24 had a spleen volume >5 MN. In 17 patients spleen volume reached a value <5 MN (71%). Median time to recovery in 17 patients was 3.26 years (range 0.13-14.55 years).

Of the patients with a QCSI measurement at the start of treatment, 13 had a value <23%. In 10 out of these 13 patients, QCSI values increased >23% during follow-up (77%). Median time to recovery in these 10 patients was 2.85 years (range 1.01-5.09 years).

For comparison with literature data we calculated response in our cohort after 2 years of ERT:

-Haemoglobin level normalized in 65%

-Platelet count >100,000/uL in 46%

-Liver volume <1.25 MN in 37%

-Spleen volume <5 MN in 42%

In general, the haemoglobin level is the first parameter to normalize after initiation of treatment. Platelet counts are slower to respond and partially dependent on the reduction in spleen volume achieved. However, reversal of cytopenia is generally seen before normalization of organ volumes, although organomegaly shows marked response already after 6 months of treatment. At baseline splenomegaly is more extensive compared to hepatomegaly and liver volume is first to normalize if at all.

Studies vary in the duration of follow-up on ERT. Studies with a shorter period of follow-up generally report lower response rates. Response in our cohort after 2 years of ERT somewhat well in line with Registry data . Response in the Dutch cohort is lower for haemoglobin level, but higher for platelet count, possibly owing to differences in cut-off values.

ERT data: complications (bone complications, Sx)

Table S1D-2C offers an overview of studies reporting on the incidence of bone complications after initiation of treatment.

Comparison to AMC data

Within the Dutch cohort four out of 29 patients naïve to bone complications develop a bone complication while treated (14%), while 2 out of 10 patients develop a second bone complication after a first bone complication (20%). Our study does not distinguish between patients that experience >2 bone complications; they remain in the phase ‘multiple complications’. Studies do not always specify whether bone complications occurred in patients with a history of bone events or not. Nonetheless our data seem to be in accordance with the literature.

Natural history and ERT data on developing complications: malignancies

Several studies report on the incidence of GD associated cancers, but a number of these were performed in Ashkenazi Jewish populations that were excluded from our comparison. A study from 2009 by Grosbois et al comments on the occurrence of gammopathy in 105 type I GD patients. Two patients within this cohort showed malignant transformation while treated. A study from the ICGG (2005) in 2510 type I patients shows that 0.4% suffered from a MM .

Comparison to AMC data

Nine patients in the Dutch cohort developed a GD associated malignancy. Four patients developed a hepatocellular carcinoma (4%), while 5 patients suffered from a multiple myeloma / amyloidosis (6%). These data are in line with the papers from Lee and Grosbois. There is a strong association between the incidence of multiple myeloma and age (2;18). Seventy percent of the patients in the Registry study are below the age of 44 years which might explain the low frequency of MM reported for this cohort .

**Table S1A. Gaucher disease states in Markov model**

| ***Disease state*** | ***Definition*** |
| --- | --- |
| **Asymptomatic** |  |
| **Signs/Symptoms** | A record of signs/symptoms, organomegaly and / or cytopenia |
| **Recovery** | Recovery was defined as the combination of all of the following criteria:  1. Haemoglobin levels >13.5 g/dl (8.4 mmol/L) in men and >12 g/dl (7.5 mmol/L) in women (American Society of Hematology);  2. Platelet count >100*10E9/L , for one year (if this was the case, then the date of the first measurement was recorded as the date of resolution);  3. Liver volume <1.25 multiples of normal (MN) and spleen volume <5 MN at two consecutive measurements ;  4. A bone marrow fat fraction Ff as assessed by Dixon’s Quantitative Chemical Shift Imaging >23% at two consecutive measurements. |
| **Splenectomy** | Splenectomy |
| **Bone complication** | Single bone complication defined as the occurrence of  -avascular necrosis  -pathological fractures  -vertebral collapse  -osteomyelitis and / or bone crises / infarctions. A bone crisis was defined as an episode of severe pain localized in a bone (no joint), requiring opioids and / or hospitalization, and / or accompanied by signs of inflammation (e.g. fever), and / or imaging abnormalities (X-ray, magnetic resonance imaging). |
| **Multiple complications** | Multiple bone complications and / or the combination of one or more bone complications, splenectomy, Parkinson disease or pulmonary hypertension |
| **Malignancy** | Multiple myeloma, amyloidosis, hepatocellular carcinoma |
| **Death** |  |

**Table S1**B-1. Yearly transition probabilities for untreated patients

| **Phase** | **Transition to** | **Yearly probability** |
| --- | --- | --- |
| **Asymptomatic** | Signs/Symptoms | 0.023 |
|  | Bone complication | 0.002 |
|  | Malignancy | * |
|  | Death | CBS |
| **Signs/Symptoms** | Splenectomy | 0.021 |
|  | Bone complication | 0.007 |
|  | Malignancy | * |
|  | Death | CBS |
| **Splenectomy** | Multiple complications | 0.053 |
|  | Malignancy | 0.03** |
|  | Death | CBS |
| **Bone complication** | Multiple complications | 0.069 |
|  | Malignancy | * |
|  | Death | CBS |
| **Multiple complications** | Malignancy | 0.016** |
|  | Death | CBS |
| **Malignancy** | Death | 0.819** |

CBS: data derived from the CBS

* Chances were assumed to be small, but not 0.

** Transition probability calculated for the treated cohort according to the base case scenario was used in the untreated cohort.

**Table S1**B-2. Yearly transition probabilities for treated patients in the base case scenario

| **Phase** | **Transition to** | **Yearly probability** |
| --- | --- | --- |
| **Signs/Symptoms** | Recovery | 0.18** |
|  | Splenectomy | * |
|  | Bone complication | 0.009 |
|  | Malignancy | * |
|  | Death | CBS |
| **Recovery** | Splenectomy | * |
|  | Bone complication | 0.004 |
|  | Malignancy | * |
|  | Death | CBS |
| **Splenectomy** | Multiple complications | 0 |
|  | Malignancy | * |
|  | Death | CBS |
| **Bone complication** | Multiple complications | 0.024 |
|  | Malignancy | * |
|  | Death | CBS |
| **Multiple complications** | Malignancy | * |
|  | Death | CBS |
| **Malignancy** | Death | 0.819 |

CBS: data derived from the CBS* Chances were assumed to be small, but not 0.

** Transition probability used as calculated according to the historical scenario.

**Table S1**B-3. Yearly transition probabilities for treated patients in the historical scenario

| **Phase** | **Transition to** | **Yearly probability** |
| --- | --- | --- |
| **Signs/Symptoms** | Recovery | 0.18 |
|  | Splenectomy | * |
|  | Bone complication | 0.035 |
|  | Malignancy | * |
|  | Death | CBS |
| **Recovery** | Splenectomy | * |
|  | Bone complication | 0.012 |
|  | Malignancy | * |
|  | Death | CBS |
| **Splenectomy** | Multiple complications | 0 |
|  | Malignancy | * |
|  | Death | CBS |
| **Bone complication** | Multiple complications | 0.042 |
|  | Malignancy | * |
|  | Death | CBS |
| **Multiple complications** | Malignancy | * |
|  | Death | CBS |
| **Malignancy** | Death | 0.819 |

CBS: data derived from the CBS

* Chances were assumed to be small, but not 0.

**Table S1C. Distribution of patients over the different disease states in the year 1991**

| **Phase** | **Historical I** | **Historical II** |
| --- | --- | --- |
| Asymptomatic | 32.1 | 0 |
| Signs/Symptoms | 31 | 45.6 |
| Splenectomy | 10.7 | 15.8 |
| Bone complication | 3.6 | 5.3 |
| Multiple complications | 22.6 | 33.3 |
| Malignancy | 0 | 0 |

**Table S1D-1.**

| **Genotype** | **Registry, 2000** | | **Zimran, 1992** | | **Giraldo, 2000** | | **AMC** | |
| --- | --- | --- | --- | --- | --- | --- | --- | --- |
|  | N (%) | Mean age at *diagnosis* | N (%) | Mean age at *diagnosis* | N (%) | Mean age at *diagnosis* | N (%) | Mean age at *symptoms* |
| N370S/N370S | 149 (23) | 27.2 | 19 (36) | 37.3 | 11 (10) | 38.7 | 6 (9) | 38.1 |
| N370S/other | 386 (60) | 15 | 27 (51) | 11.3 | 75 (66) | 24.6 | 57 (81) | 25,8 |
| other/other | 109 (17) | 5.9* | 7 (13) | 6.8 | 28 (25) | 18.6 | 7 (10) | 14.3 |
|  | 644 | 17.4 | 53 | 25 (N=50) | 114 | 24 | 70 | 25.7 |

* Value recalculated from data reported in the article.

**Table S1D-2A. Percentages and age at occurrence of major complications in the pre ERT era; only studies on GD** I patients with a comparable genetic background are included

| **Author** | **Year** | **N** | **Specifics** | **Mean age** | **Mean age at diagnosis / symptoms** | **Sx (%)** | **Bone pain (%)** | **Bone complications (%)** | **Malignancies (%)** |
| --- | --- | --- | --- | --- | --- | --- | --- | --- | --- |
| Reich | 1951 | 20 | Adults | 40.4 |  | 20 | 60 | 50* |  |
| Lee | 1982 | 239 |  |  |  | 48 |  | 41 | N=35: 9 MM, 3 HCC |
| Rodrigue | 1999 | 51 | Patients evaluated for ERT trial | 37 | 13.1 / ? |  | 69 | Osteonecrosis 29 |  |
| Drugan | 2002 | 20 |  | 24.2 |  | 45 | 65 | AVN, fractures 25 |  |
| Revest (14) | 2009 | 12 | N=1 type II patient | 48.7 | 31.1 / 32.3 | ? | 27 |  |  |
| Charrow *Registry* | 2000 | 1698 | Includes GD II/III patients | 34.7 | 17.4 / ? | 28 |  | AVN 25  Fractures 15  Infarction 25  Crises 35 |  |

* Radiological evidence of GD.

**Table S1D-2B. Studies on the effect of ERT, only papers in which individual responses could be assessed are included**

| **Author** | **Year** | **Nr. of patients** | **Specifics** | **Recovery haemoglobin** | **Recovery platelets** | **Recovery organomegaly** |
| --- | --- | --- | --- | --- | --- | --- |
| Barton | 1991 | 12 (8 adults) | Max. follow-up 1 year, 120U/kg/m  No Sx | 1/5 adults (20%) | 2/7 (29%) | Liver 0/11  Spleen 0/11 |
| Figueroa | 1992 | 14 | 6 months, 30-120U/kg/m  10 Sx |  | 1/4 (25%) | Liver 3/13 (23%)  Spleen 0/4 |
| Fallet | 1992 | 11 (8 adults) | >6 months, 60-120U/kg/m  4 Sx | 6/8 adults (75%) | 4/6 (67%) | Liver 0/11  Spleen 0/7 |
| Pastores* | 1993 | 33 (24 adults) | 6-24 months, 60-120U/kg/m  15 Sx | 10/30 |  | Liver 4/28 (14%)  Spleen 1/18 (6%) |
| Zimran | 1994 | 29 (20 adults) | 6-28 months, 30U/kg/m  15 Sx | 7/16 adults (44%) | 8/10 (80%) |  |
| Bembi | 1994 | 9 (3 adults) | 12-24 months, 30-60U/kg/m  4 Sx | 1/3 adults (33%) | 1/2 (50%) | Liver 5/8 (63%)  Spleen 1/4 |
| Niederau | 1994 | 5 adults | 12-18 months, 40-100U/kg/m | 4/4 | 2/4 (50%) |  |
| Grabowski | 1995 | 30 | 6-9 months, 120U/kg/m  Ceredase vs Cerezyme, no Sx | 11/21 adults (52%) | 6/25 (24%) | Liver 3/25 (12%)  Spleen 2/29 (7%) |
| Beutler | 1995 | 32 | 6-42 months, 30U/kg/m  17 Sx |  | 6/10 (60%) |  |
| Elstein | 1998 | 28 adults | 6-24 months, 30U/kg/m  3 Sx | 11/22 (50%) | 4/22 (18%) |  |
| Perez-Calvo** | 1997 |  | 1-year | 82,3% | 47% |  |
| Mrsic*** | 2003 | 4 | 1-year, 60-120U/kg/m | 100% | 100% | Liver 0/4  Spleen 0/4 |
| Goldblatt**** | 2005 | 48 (29 adults) | 6-114 months, 60-120U/kg/m  11 Sx, 6 type III | 85%  78% after 24 months | 75%  65% after 24 months |  |
| Wilson***** | 2007 | 12 (11 adults) | 2-5 years, 30U/kg/m  4 Sx | 6/6 | 5/6 (83%) | Liver 8/10 (80%)  Spleen 8/8 (80%) |
| Weinreb *Registry******* | 2002 | 1028 | 2-5 years | 110/135 non-Sx (81%) | 89/222 non-Sx (40%) | Liver 49/94 (52%)  Spleen 24/96 (25%) |

* Haemoglobin level, hepatomegaly, splenomegaly normalized, no criteria stated.

** Haemoglobin level, platelet counts normal, no criteria stated.

*** No criteria stated for haemoglobin level, organomegaly.

**** Criteria for normal haemoglobin level differ slightly, platelet count >150*10E9/L.

***** Haemoglobin level >13.5 g/dL in males, >11.5 g/dL in females, Liver <1.5MN, Spleen <8 MN.

****** Criteria for normal haemoglobin level differ slightly, platelet count >120.

**Table S1D-2C. Studies describing the effect of ERT on bone complications**

| **Author** | **Year** | **Nr. of patients** | **Specifics** | **% Sx** | **% of bone complications pre-ERT** | **% of bone complications post-ERT** |
| --- | --- | --- | --- | --- | --- | --- |
| Beutler | 1995 | 30 | Children and adults  Follow-up 6-43 months ERT  30-120U/kg/m | 57 |  | 4/30 (13%) |
| Belmatoug | 1995 | 45 | Children and adult, severely affected  Follow-up 14 months ERT  120U/kg/m | 33 | 58%* | No new fractures / osteonecrosis |
| Elstein | 1996 | 14 | Adults, severe skeletal involvement at t=0  Follow-up 24-48 months ERT  30U/kg/m | 57 | 100% | 1/14 (7%) |
| Schiffmann | 2002 | 29 | Adults  Follow-up 24 months ERT  60U/kg/m | 100 |  | 2/29 AVN (7%)  7/29 bone crises (24%) |
| Sims | 2008 | 33 | 32 Adults  Follow-up 48 months ERT120U/kg/m | 15 | 39% bone crisis  6% fractures  36% medullary infarct.  6% osteoarticular necrosis  36% lytic lesions | 9% bone crises  9% fractures  12% medullary infarct.  15% osteoarticular necrosis  9% lytic lesions |
| Stirnemann | 2010 | 62 | Children and adults  4 type III patients  Follow-up max 15 years ERT  30-120U/kg/m | 34 | 47% | 16% |
| Deegan | 2011 | 100 | Adults  4 type III patients  3 months-16 years ERT | 44 | 35% osteonecrosis | 9% osteonecrosis |
| Weinreb *Registry* | 2002 | 176 | Follow-up 2 years of ERT |  | 29% bone crisis | 13% |
| Charrow *Registry* | 2007 | 219 | Incidence of bone crisis 1 year prior to start ERT vs 1 year post start ERT |  | 17% bone crisis | 5% bone crisis |
| Mistry  *Registry* | 2009 | 2700 | Occurrence of AVN on ERT in patients with a negative history for AVN | 22 (incl partial) | 0% AVN | 8% AVN |

* Plain radiologic signs or symptoms of skeletal involvement.

**Table S1E-1**.

|  |  | **AMC – ‘Recovery’** | **Registry- Therapeutic Goals** |
| --- | --- | --- | --- |
| **Haemoglobin** | Male, adult | >13.5 g/dL | >12 g/dL |
|  | Female, adult | >12 g/dL | >11 g/dL |
| **Platelets** |  | >100,000/uL | >120,000/uL, unless platelet count was <60,000/uL at baseline |
| **Liver volume** |  | <1,25 MN | <1,5 MN |
| **Spleen volume** |  | <5 MN | <8 MN |
| **Bone pain** |  | NA | none or very mild |
| **Bone crises** |  | NA | none |
| **QCSI** |  | >23% | NA |

1. van Dussen L, Biegstraaten M, Dijkgraaf MG, et al. Modelling Gaucher disease progression: long term enzyme replacement therapy reduces the incidence of splenectomy and bone complications, 2013.

2. Lee RE. The pathology of Gaucher disease. ProgClinBiolRes 1982;**95**:177-217

3. Kyle RA, Therneau TM, Rajkumar SV, et al. A long-term study of prognosis in monoclonal gammopathy of undetermined significance. NEnglJMed 2002;**346**(8):564-69 doi: 10.1056/NEJMoa01133202 [doi];346/8/564 [pii][published Online First: Epub Date]|.

4. Brady K, Corash L, Bhargava V. Multiple myeloma arising from monoclonal gammopathy of undetermined significance in a patient with Gaucher's disease. ArchPatholLab Med 1997;**121**(10):1108-11

5. de Fost M, Out TA, de Wilde FA, et al. Immunoglobulin and free light chain abnormalities in Gaucher disease type I: data from an adult cohort of 63 patients and review of the literature. AnnHematol 2008;**87**(6):439-49

6. Ponce E, Moskovitz J, Grabowski G. Enzyme therapy in Gaucher disease type 1: effect of neutralizing antibodies to acid beta-glucosidase. Blood 1997;**90**(1):43-48

7. Krasnewich D, Dietrich K, Bauer L, et al. Splenectomy in Gaucher disease: new management dilemmas. Blood 1998;**91**(8):3085-87

8. Charrow J, Andersson HC, Kaplan P, et al. The Gaucher registry: demographics and disease characteristics of 1698 patients with Gaucher disease. ArchInternMed 2000;**160**(18):2835-43 doi: ioi90854 [pii][published Online First: Epub Date]|.

9. Zimran A, Kay A, Gelbart T, et al. Gaucher disease. Clinical, laboratory, radiologic, and genetic features of 53 patients. Medicine (Baltimore) 1992;**71**(6):337-53

10. Giraldo P, Pocovi M, Perez-Calvo J, et al. Report of the Spanish Gaucher's disease registry: clinical and genetic characteristics. Haematologica 2000;**85**(8):792-99

11. Drugan C, Procopciuc L, Jebeleanu G, et al. Gaucher disease in Romanian patients: incidence of the most common mutations and phenotypic manifestations. EurJHumGenet 2002;**10**(9):511-15 doi: 10.1038/sj.ejhg.5200845 [doi][published Online First: Epub Date]|.

12. Reich C, SEIFE M, Kessler BJ. Gaucher's disease: a review, and discussion of twenty cases. Medicine (Baltimore) 1951;**30**(1):1-20

13. Rodrigue SW, Rosenthal DI, Barton NW, et al. Risk factors for osteonecrosis in patients with type 1 Gaucher's disease. ClinOrthopRelat Res 1999(362):201-07

14. Revest M, Perlat A, Decaux O, et al. [Gaucher's disease in Rennes University hospital: a 10-year retrospective study]. RevMedInterne 2009;**30**(10):847-56 doi: S0248-8663(09)00513-X [pii];10.1016/j.revmed.2009.03.357 [doi][published Online First: Epub Date]|.

15. Weinreb N, Taylor J, Cox T, et al. A benchmark analysis of the achievement of therapeutic goals for type 1 Gaucher disease patients treated with imiglucerase. AmJHematol 2008;**83**(12):890-95

16. Pastores GM, Weinreb NJ, Aerts H, et al. Therapeutic goals in the treatment of Gaucher disease. SeminHematol 2004;**41**(4 Suppl 5):4-14

17. Weinreb NJ, Charrow J, Andersson HC, et al. Effectiveness of enzyme replacement therapy in 1028 patients with type 1 Gaucher disease after 2 to 5 years of treatment: a report from the Gaucher Registry. AmJMed 2002;**113**(2):112-19

18. Grosbois B, Rose C, Noel E, et al. Gaucher disease and monoclonal gammopathy: a report of 17 cases and impact of therapy. Blood Cells MolDis 2009;**43**(1):138-39 doi: S1079-9796(09)00102-8 [pii];10.1016/j.bcmd.2009.04.002 [doi][published Online First: Epub Date]|.

19. Rosenbloom BE, Weinreb NJ, Zimran A, et al. Gaucher disease and cancer incidence: a study from the Gaucher Registry. Blood 2005;**105**(12):4569-72 doi: 2004-12-4672 [pii];10.1182/blood-2004-12-4672 [doi][published Online First: Epub Date]|.

20. Neunert C, Lim W, Crowther M, et al. The American Society of Hematology 2011 evidence-based practice guideline for immune thrombocytopenia. Blood 2011;**117**(16):4190-207 doi: blood-2010-08-302984 [pii];10.1182/blood-2010-08-302984 [doi][published Online First: Epub Date]|.

21. Barton NW, Brady RO, Dambrosia JM, et al. Replacement therapy for inherited enzyme deficiency--macrophage-targeted glucocerebrosidase for Gaucher's disease. NEnglJMed 1991;**324**(21):1464-70

22. Figueroa ML, Rosenbloom BE, Kay AC, et al. A less costly regimen of alglucerase to treat Gaucher's disease. NEnglJMed 1992;**327**(23):1632-36

23. Fallet S, Grace ME, Sibille A, et al. Enzyme augmentation in moderate to life-threatening Gaucher disease. PediatrRes 1992;**31**(5):496-502

24. Pastores GM, Sibille AR, Grabowski GA. Enzyme therapy in Gaucher disease type 1: dosage efficacy and adverse effects in 33 patients treated for 6 to 24 months. Blood 1993;**82**(2):408-16

25. Zimran A, Elstein D, Kannai R, et al. Low-dose enzyme replacement therapy for Gaucher's disease: effects of age, sex, genotype, and clinical features on response to treatment. AmJMed 1994;**97**(1):3-13

26. Bembi B, Zanatta M, Carrozzi M, et al. Enzyme replacement treatment in type 1 and type 3 Gaucher's disease. Lancet 1994;**344**(8938):1679-82

27. Niederau C, Holderer A, Heintges T, et al. Glucocerebrosidase for treatment of Gaucher's disease: first German long-term results. JHepatol 1994;**21**(4):610-17 doi: S0168-8278(94)80109-6 [pii][published Online First: Epub Date]|.

28. Grabowski GA, Barton NW, Pastores G, et al. Enzyme therapy in type 1 Gaucher disease: comparative efficacy of mannose-terminated glucocerebrosidase from natural and recombinant sources. AnnInternMed 1995;**122**(1):33-39

29. Beutler E, Demina A, Laubscher K, et al. The clinical course of treated and untreated Gaucher disease. A study of 45 patients. Blood Cells MolDis 1995;**21**(2):86-108

30. Elstein D, Abrahamov A, Hadas-Halpern I, et al. Low-dose low-frequency imiglucerase as a starting regimen of enzyme replacement therapy for patients with type I Gaucher disease. QJM 1998;**91**(7):483-88

31. Perez-Calvo JI, Giraldo P, Giralt M. [Alglucerase treatment of type I Gaucher's disease. Preliminary results in Spain. Spanish Group on Gaucher's Disease]. Sangre (Barc) 1997;**42**(3):189-94

32. Mrsic M, Stavljenic-Rukavina A, Fumic K, et al. Management of Gaucher disease in a post-communist transitional health care system: Croatian experience. CroatMedJ 2003;**44**(5):606-09

33. Goldblatt J, Szer J, Fletcher JM, et al. Enzyme replacement therapy for Gaucher disease in Australia. InternMedJ 2005;**35**(3):156-61 doi: IMJ765 [pii];10.1111/j.1445-5994.2004.00765.x [doi][published Online First: Epub Date]|.

34. Wilson C, Spearing R, Teague L, et al. The outcome of clinical parameters in adults with severe Type I Gaucher disease using very low dose enzyme replacement therapy. MolGenetMetab 2007;**92**(1-2):131-36 doi: S1096-7192(07)00191-6 [pii];10.1016/j.ymgme.2007.05.013 [doi][published Online First: Epub Date]|.

35. Belmatoug N, T BdV. Skeletal Response to Enzyme Replacement Therapy for Type I Gaucher Disease: A Preliminary Report of the French Experience. SeminHematol 1995;**32**(3, Suppl. 1):33-38

36. Elstein D, Hadas-Halpern I, Itzchaki M, et al. Effect of low-dose enzyme replacement therapy on bones in Gaucher disease patients with severe skeletal involvement. Blood Cells MolDis 1996;**22**(2):104-11

37. Schiffmann R, Mankin H, Dambrosia JM, et al. Decreased bone density in splenectomized Gaucher patients receiving enzyme replacement therapy. Blood Cells MolDis 2002;**28**(2):288-96

38. Sims KB, Pastores GM, Weinreb NJ, et al. Improvement of bone disease by imiglucerase (Cerezyme) therapy in patients with skeletal manifestations of type 1 Gaucher disease: results of a 48-month longitudinal cohort study. ClinGenet 2008;**73**(5):430-40

39. Stirnemann J, Belmatoug N, Vincent C, et al. Bone events and evolution of biologic markers in Gaucher disease before and during treatment. Arthritis ResTher 2010;**12**(4):R156 doi: ar3111 [pii];10.1186/ar3111 [doi][published Online First: Epub Date]|.

40. Deegan PB, Pavlova E, Tindall J, et al. Osseous manifestations of adult Gaucher disease in the era of enzyme replacement therapy. Medicine (Baltimore) 2011;**90**(1):52-60 doi: 10.1097/MD.0b013e3182057be4 [doi];00005792-201101000-00005 [pii][published Online First: Epub Date]|.

41. Charrow J, Dulisse B, Grabowski GA, et al. The effect of enzyme replacement therapy on bone crisis and bone pain in patients with type 1 Gaucher disease. ClinGenet 2007;**71**(3):205-11

42. Mistry PK, Deegan P, Vellodi A, et al. Timing of initiation of enzyme replacement therapy after diagnosis of type 1 Gaucher disease: effect on incidence of avascular necrosis. BrJHaematol 2009;**147**(4):561-70 doi: BJH7872 [pii];10.1111/j.1365-2141.2009.07872.x [doi][published Online First: Epub Date]|.
